# Cohort Removal Induces Changes in Body Temperature, Pain Sensitivity, and Anxiety-Like Behavior

**DOI:** 10.3389/fnbeh.2016.00099

**Published:** 2016-06-03

**Authors:** Keizo Takao, Hirotaka Shoji, Satoko Hattori, Tsuyoshi Miyakawa

**Affiliations:** ^1^Section of Behavior Patterns, Center for Genetic Analysis of Behavior, National Institute for Physiological SciencesOkazaki, Japan; ^2^Japan Science and Technology Agency, Core Research for Evolutional Science and Technology, CRESTKawaguchi, Japan; ^3^Division of Animal Resources and Development, Life Science Research Center, University of ToyamaToyama, Japan; ^4^Division of Systems Medical Science, Institute for Comprehensive Medical Science, Fujita Health UniversityToyoake, Japan

**Keywords:** mouse behavior, stress, cohort removal, anxiety-like behavior, rectal tempetature, pain sensitivity, corticosterone

## Abstract

Mouse behavior is analyzed to elucidate the effects of various experimental manipulations, including gene mutation and drug administration. When the effect of a factor of interest is assessed, other factors, such as age, sex, temperature, apparatus, and housing, are controlled in experiments by matching, counterbalancing, and/or randomizing. One such factor that has not attracted much attention is the effect of sequential removal of animals from a common cage (cohort removal). Here we evaluated the effects of cohort removal on rectal temperature, pain sensitivity, and anxiety-like behavior by analyzing the combined data of a large number of C57BL/6J mice that we collected using a comprehensive behavioral test battery. Rectal temperature increased in a stepwise manner according to the position of sequential removal from the cage, consistent with previous reports. In the hot plate test, the mice that were removed first from the cage had a significantly longer latency to show the first paw response than the mice removed later. In the elevated plus maze, the mice removed first spent significantly less time on the open arms compared to the mice removed later. The results of the present study demonstrated that cohort removal induces changes in body temperature, pain sensitivity, and anxiety-like behavior in mice. Cohort removal also increased the plasma corticosterone concentration in mice. Thus, the ordinal position in the sequence of removal from the cage should be carefully counterbalanced between groups when the effect of experimental manipulations, including gene manipulation and drug administration, are examined using behavioral tests.

## Introduction

Mice are subjected to behavioral tests in laboratories to examine the effects of gene mutations, drug treatments, and other experimental manipulations on brain function. In characterizing mouse behavior, various factors must be taken into account in addition to the main effects under study. The impact of those factors on the analyses should be minimized in the experiments by matching, counterbalancing, and/or randomizing. Although some of these factors have not drawn much attention of the researchers in the field, their effects should not be ignored. One such factor is the sequential removal of animals from a common cage, which is also known as “cohort removal”. In group-housed mice, those removed last from the home cage have a higher rectal temperature than those removed first (Borsini et al., 1989; Rodgers et al., 1994), which is referred to as stress-induced hyperthermia. Rodgers et al. also examined the effect of cohort removal on anxiety-like behavior in the elevated plus maze test (Rodgers et al., 1994) and reported that cohort removal does not influence any behaviors measured in that test. The effect of cohort removal on anxiety-like behaviors of mice assesed in the elevated plus maze, however, may not be large enough to detect using a small number of subjects. In the present study, we combined data collected using our behavioral test battery to examine the effect of cohort removal on mouse behavior with a much larger number of subjects.

Since 2003, our research group has examined behavioral phenotypes of 170 strains of genetically-engineered mice using what we call a “comprehensive behavioral test battery” (Takao et al., 2007). When we characterize the behavioral phenotypes of mutant mice using the test battery, their wild-type littermates are often used as controls. The C57BL/6J strain is one of the most commonly used background strains in this research field. Thus, we have collected behavioral data from 1910 control wild-type C57BL/6J male mice. We obtained the data using the same, standardized protocols, and the data are therefore suitable for combined data analyses (Matsuo et al., 2010).

In our behavioral test battery, to increase the efficiency of screening behavioral phenotypes, most of the tests are conducted with four or more (up to eight) mice at a time. In those tests, the effect of cohort removal may not be important because the mice are all removed from their cages essentially at the same time. On the other hand, in tests performed with a single apparatus, the mice are removed one by one due to the limitations imposed by the apparatus or experimenter. In our behavioral battery, the rectal temperature measurement, hot plate test, and elevated plus maze test are conducted in a way that requires the mice to be removed from their cage sequentially and tested using a single apparatus. Therefore, in the present study, we investigated how the order of removal affects rectal temperature, pain sensitivity in the hot plate test, and anxiety-like behavior in the elevated plus maze.

## Materials and Methods

### Animals

Behavioral data of control wild-type C57BL/6J male mice that we collected from a comprehensive behavioral analysis of 80 strains of genetically engineered mice or experimentally treated mice were used. Data of wild-type control mice with a C57BL/6J background were extracted and used for the combined analyses presented in this study. Information of each mouse and the behavioral data analyzed in this study, including age, vendor, donor strain, and dates when the experiments were conducted, are available through the Mouse Phenotype Database^1^.

We excluded no behavioral data except for some specific cases in which the animals fell off of the arms of the elevated plus maze test, or the movies were not recorded due to technical problems. Behavioral data without information on the order of cohort removal were excluded from the analyses. For subjects used for the analysis, more than 80% of the mice used were backcrossed at least six times (and more than 95% of the mice used were backcrossed at least five times) with C57BL/6J mice. Mice housed singly or more than six per cage were excluded from the analyses. Mice ranged in age from 40 to 725 days (rectal temperature), 71–412 days (hot plate test), and 60–407 days (elevated plus maze test). Numbers of mice are indicated in the figures. Behavioral testing was performed between 9:00 a.m. and 6:00 p.m.

Our behavioral test battery includes assessment of general health, light/dark transition, open field, elevated plus maze, rotarod, hot plate, social interaction, prepulse inhibition, Porsolt forced swim, and contextual fear conditioning tests. Each experiment was performed in the order indicated. The interval between each experiment was at least 1 day. After the tests, all apparatuses were cleaned with super hypochlorous water to prevent a bias due to olfactory cues. All behavioral testing procedures were approved by the Animal Care and Use Committee of Kyoto University Graduate School of Medicine, the Animal Care and Use Committee of Fujita Health University, and the Animal Research Committee of the National Institute for Physiological Sciences.

### Rectal Temperature Measurement

Measurement of rectal temperature in mice was performed in the breeding room to avoid possible additional stressful factors. Mice were arbitrarily picked up without identification and the probe of a BAT-12 Microprobe Thermometer (Physitemp Instruments, Clifton, NJ, USA), which was immersed in mineral oil before use, was placed approximately 10 mm into the rectal canal after lifting the tail to measure the rectal temperature. During the procedure, the mice were placed on a metal grid. Mouse identification was performed after the measurement of each subject. After the measurement, the mice were placed in a holding cage until all the mice from the same home cage had completed the test.

### Hot Plate Test

The hot plate test was used to evaluate sensitivity to a painful stimulus. As previously described (Takao et al., 2010), the mice were placed on a 55.0 (±0.3)°C hot plate (Columbus Instruments, Columbus, OH, USA), and the latency to show the first hind-paw response was recorded. The hind-paw response was defined as either a foot shake or a paw lick. After the measurement, mice were placed in a holding cage until all the mice from the same home cage were subjected to the test. Mice removed from the cage in the fifth position were excluded from the analyses due to the insufficient number of subjects.

### Elevated Plus Maze Test

The elevated plus maze test was conducted as previously described (Komada et al., 2008). The elevated plus maze (O’Hara and Co., Tokyo, Japan) comprised two open arms (25 cm × 5 cm) and two enclosed arms of the same size, with 15-cm high transparent walls. The arms and central square were constructed of white plastic plates and were elevated to a height of 55 cm above the floor. To minimize the likelihood of animals falling from the apparatus, 3-mm high plastic ledges surrounded the open arms. Arms of the same type were arranged at opposite sides to each other. Each mouse was placed in the central square of the maze (5 cm × 5 cm), facing one of the enclosed arms. Mice removed from the cage fifth were excluded from the analyses because the number of mice was insufficient. The level of lighting at the surface of the maze was 100 lux. Mouse behavior was recorded during a 10-min test period. The numbers of entries into, and the time spent on the open and enclosed arms, were recorded. After the measurement, the mice were placed in a holding cage until all the mice from the same home cage were subjected to the test. For data analysis, we used the following four measures: percentage of entries into the open arms, percentage of time spent on the open arms, number of total arm entries, and total distance traveled (cm). Data acquisition and analysis were performed automatically using ImageEP software. The software was based on the public domain NIH Image program (developed at the USA National Institutes of Health and available on the Internet^2^) and ImageJ program, which was modified for the test by the authors. ImageEP software (Komada et al., 2008) is freely available in the URL^3^.

### Plasma Corticosterone Concentration Measurement

Cages containing mice were transferred to an experimental room and left undisturbed for 6 h per day for 5 days to habituate the subjects to the environment and handling prior to blood collection. On the 6th day, the cages were transferred to the experimental room, and after the last habituation for 2 h the mice were removed from the cages at 15-min intervals in sequential order. The mice were immediately transferred to another room and decapitated for blood collection. Blood samples were collected in heparinized tubes and kept on ice. Blood was centrifuged at 3000× g for 10 min at 4°C, and plasma was removed and stored at −80°C until use. Plasma corticosterone was measured using an enzyme immunoassay kit following the manufacturer’s protocol (Assay Designs; Enzo Life Sciences, Farmingdale, NY, USA).

### Statistical Analysis

Statistical analysis was conducted using SAS University Edition (SAS Institute, Cary, NC, USA). Data were analyzed by one-way ANOVA followed by Fisher’s PLSD multiple comparison tests. Significance levels of multiple comparisons were controlled using an FDR procedure *q* = 0.05; Benjamini and Hochberg, 1995; Verhoeven et al., 2005). Values in graphs are expressed as mean ± SEM.

## Results

### Cohort Removal Increased Rectal Temperature

**Figure 1:**
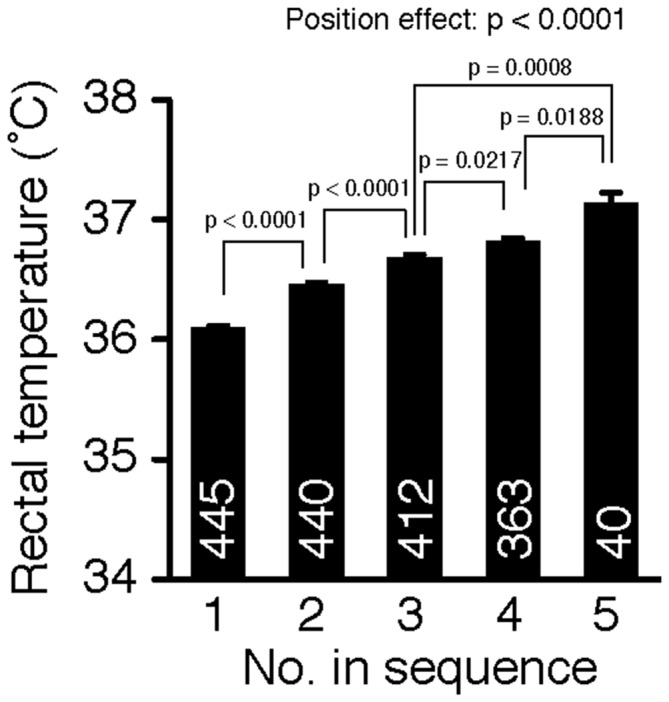
**Cohort removal induced hyperthermia in mice**. Rectal temperature was compared among mice removed in each position of sequential removal. Cohort removal of mice from a group-housed cage induced hyperthermia. Rectal temperature increased in a stepwise manner with increments in the position of removal.

The order of the sequential removal from the home cage significantly influenced the rectal temperature of mice (Figure 1, one-way ANOVA, position effect, F_(4,1695)_ = 52.29, *p* < 0.0001). *Post hoc* tests using Fisher’s PLSD revealed that the mice removed earlier had a lower rectal temperature than mice removed later (position 1 vs. position 2: *p* < 0.0001, position 2 vs. position 3: *p* < 0.0001, position 3 vs. position 4: *p* = 0.0217, position 4 vs. position 5: *p* = 0.0188). Rectal temperature increased significantly in a stepwise manner according to the position of removal. The significance of these differences among the positions all survived the Benjamini and Hochberg false discovery rate (FDR) procedure (Benjamini-Hochberg Critical Value; *q* = 0.05; Benjamini and Hochberg, 1995; Verhoeven et al., 2005). These results suggest that cohort removal induced hyperthermia in mice in a manner dependent on the number of mice removed from their cage before the test. On the other hand, body weight was not significantly affected by the order of removal (one-way ANOVA, position effect, F_(4,1695)_ = 2.29, *p* = 0.0575).

We performed an analysis of covariance (ANCOVA) with age (in days) as a covariate, because the age of subjects of the present study ranged from 40 to 725 days old. This analysis revealed a significant age effect and a significant age × position interaction on rectal temperature (age effect, *p* < 0.0001; position effect, *p* < 0.0001; age × position, *p* = 0.0001), suggesting that the effect of cohort removal differs depending on the age of the subject. We then analyzed the effect of cohort removal on rectal temperature in younger mice (<200 days old) and older mice (≥200 days old) separately. Interestingly, in the old mice, cohort removal had no significant effect on rectal temperature (Supplementary Figure 1A, one-way ANOVA, position effect, F_(3,179)_ = 0.67, *p* = 0.5722), whereas in young mice cohort removal did have a significant effect on rectal temperature (Supplementary Figure 1B, one-way ANOVA, position effect, F_(3,1473)_ = 67.64, *p* < 0.0001). These results suggest that old mice are not sensitive to the effect of cohort removal stress compared to young mice.

### Cohort Removal Increased Pain Sensitivity Assessed in the Hot Plate Test

**Figure 2:**
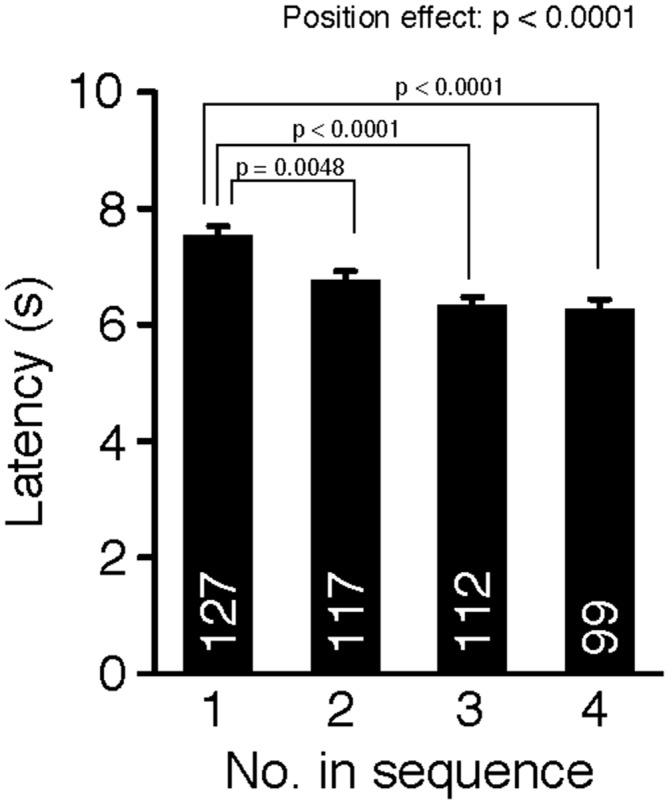
**Cohort removal increased pain sensitivity in the hot plate test**. Latencies to the first paw responses were compared among mice removed in each position of removal. Mice were sensitized to heat-pain by sequential removal from their home cage.

Cohort removal also affected pain sensitivity measured in the hot plate test (Figure 2, one-way ANOVA, position effect, F_(3,451)_ = 9.04, *p* < 0.0001). *Post hoc* tests using Fisher’s PLSD indicated that the mice removed first (position 1) had significantly longer response latencies than mice removed later (position 1 vs. positions 2, 3, or 4: *p* = 0.0048, *p* < 0.0001, *p* < 0.0001, respectively; significant at *q* = 0.05), which suggests that cohort removal increased pain sensitivity in mice. We also performed ANCOVA with age (in days) as a covariate, but there was no significant age × position interaction in the latency (age effect, *p* = 0.0676; position effect, *p* = 0.0939; age × position, *p* = 0.3559).

### Cohort Removal Altered Anxiety-Like Behavior Measured in the Elevated Plus Maze

**Figure 3:**
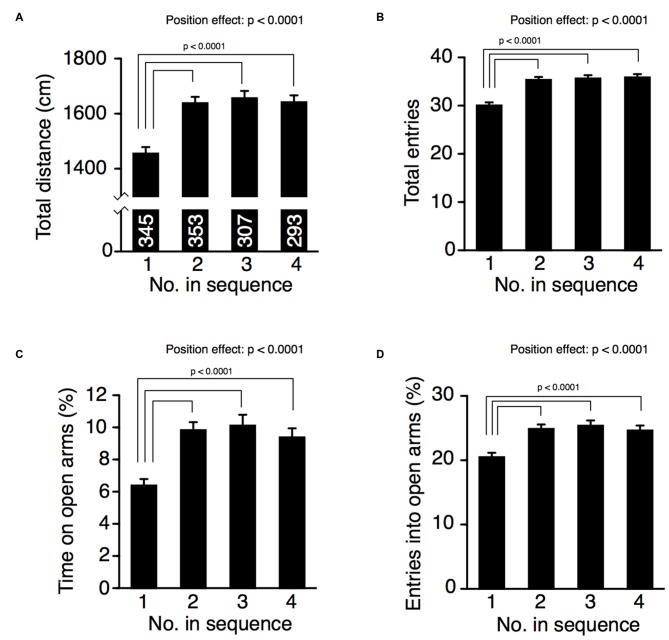
**Cohort removal altered anxiety-like behavior in the elevated plus maze test**. Total distance traveled **(A)**, total number of arm entries **(B)**, time spent on open arms **(C)**, and percentage of entries into open arms **(D)** were compared among mice at each position of removal. Mice removed second or later in the sequence exhibited increased locomotor activity and decreased anxiety-like behavior compared to mice removed first.

In the elevated plus maze, the position of removal significantly increased total distance traveled (Figure 3A, one-way ANOVA, position effect, F_(3,1294)_ = 15.98, *p* < 0.0001), number of total arm entries (Figure 3B, one-way ANOVA, position effect, F_(3,1294)_ = 18.80, *p* < 0.0001), time spent on open arms (Figure 3C, one-way ANOVA, position effect, F_(3,1294)_ = 10.77, *p* < 0.0001), and percentage of entries into open arms (Figure 3D, one-way ANOVA, position effect, F_(3,1294)_ = 9.77, *p* < 0.0001). Mice in position 1 traveled a shorter distance in the maze (Fisher’s PLSD; position 1 vs. positions 2, 3, or 4: *p*’s < 0.0001; significant at *q* = 0.05). Number of total entries in mice of position 1 was smaller than that of mice in positions 2, 3, or 4 (Fisher’s PLSD; position 1 vs. positions 2, 3, or 4: *p*’s < 0.0001; significant at *q* = 0.05). These results suggest that cohort removal induced hyperactivity in mice. Because mice instinctively tend to avoid the open arms and remain on the closed arms, the time spent on the open arms is considered an index of anxiety-like behavior (Rodgers and Dalvi, 1997). Mice removed first from their cage (position 1) spent less time on the open arms compared to mice removed later (Fisher’s PLSD; position 1 vs. positions 2, 3, or 4: *p*’s < 0.0001; significant at *q* = 0.05). We observed similar results in the number of entries onto open arms. Mice in position 1 entered the open arms less often compared to mice removed later (Fisher’s PLSD, position 1 vs. positions 2, 3, or 4: *p*’s < 0.0001; significant at *q* = 0.05). These findings suggest that cohort removal affected anxiety-like behavior in mice. We also performed ANCOVA with age (in days) as a covariate, but there was no significant age × position interaction in those four indices (age × position, total distance, *p* = 0.4884; total arm entries, *p* = 0.3216; time spent on open arms, *p* = 0.6213; entries into open arms *p* = 0.6739).

### Cohort Removal Increased Plasma Corticosterone Concentration

**Figure 4:**
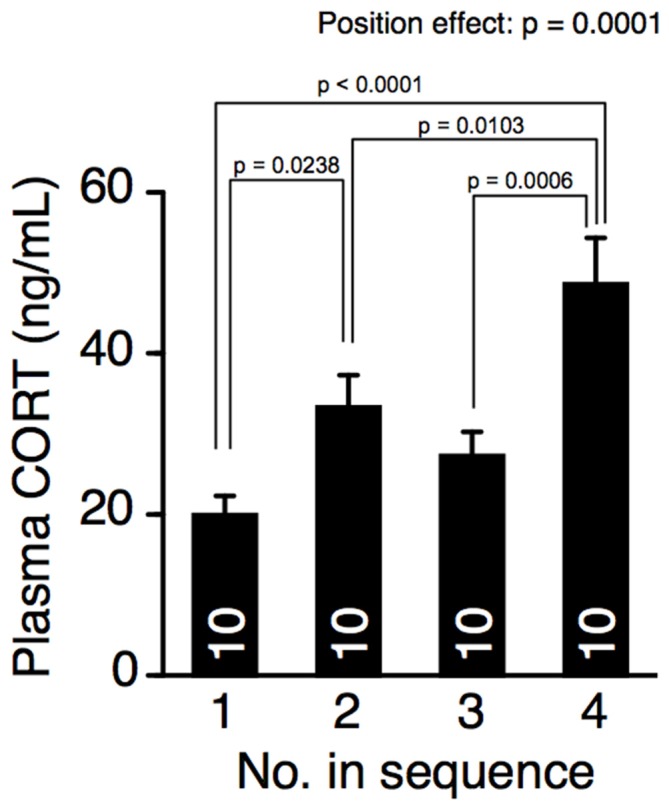
**Cohort removal increased the plasma corticosterone concentration**. Blood plasma corticosterone concentration in mice successively taken from their home cage. Corticosterone concentration in the mice of position 4 was significantly higher than that in mice removed earlier.

The effect of cohort removal on plasma corticosterone concentration was examined using a different group of 12-week-old male mice that had not been tested in the behavioral battery. The mice were removed from their home cage every 15 min and decapitated in another experimental room to prevent exposing the mice remaining in the home cages to the odors or sounds of the decapitation. The interval time was matched to that of the elevated plus maze test. The position of removal had a significant effect on plasma corticosterone concentration (Figure 4, one-way ANOVA, position effect, F_(3,36)_ = 9.261, *p* = 0.0001). Plasma corticosterone concentrations in the mice removed fourth were significantly higher than in mice removed earlier (Fisher’s PLSD, position 4 vs. positions 1, 2, or 3, *p* < 0.0001, *p* = 0.0103, *p* = 0.0006, respectively; significant at *q* = 0.05), suggesting that cohort removal increased the plasma corticosterone concentration.

## Discussion

In the present study, we performed combined data analyses to elucidate the effect of sequential removal of group-housed C57BL/6J mice on rectal temperature and behaviors in the hot plate test and the elevated plus maze test. The data were obtained from experiments using the same, standardized protocols and the same apparatuses, although the vendor, age, breeding environment, and testing dates of the mice, as well as the experimenters, which could potentially affect the results, were not necessarily the same. We used behavioral data of control wild-type C57BL/6J mice of genetically engineered mice or experimentally treated mice. In the genetically modified mice, the flanking genes from the donor strain remain in the vicinity of the target gene (Vanden Berghe et al., 2015). However, in wild-type littermates of the mutant mice, the alleles that correspond to the mutated gene are wild-type alleles. Therefore, flanking gene problem should not have markedly affected the variation of the data analyzed in the present study. Based on our combined analyses, we demonstrated that cohort removal induced hyperthermia, increased pain sensitivity, and decreased anxiety-like behavior in group-housed laboratory mice.

Previous studies reported that cohort removal induces hyperthermia in rodents (Borsini et al., 1989; Rodgers et al., 1994; Zethof et al., 1994). This phenomenon is also referred to as stress-induced hyperthermia, and it is considered to be due to anticipatory anxiety (Borsini et al., 1989; Rodgers et al., 1994; Zethof et al., 1994). In those studies, the effect of cohort removal on rectal temperature was assessed using a relatively small number of animals. Consequently, the investigators detected a difference only between those removed last and those removed first (Borsini et al., 1989; Zethof et al., 1994), or between groups removed later and earlier (Rodgers et al., 1994). In the present study, the combined data from our behavioral test battery enabled us to evaluate the effect of cohort removal on rectal temperature in more detail. The analysis presented in this study revealed that rectal temperature depended on the number of mice removed before the measurement. In general, psychological stressors induce an elevation in body temperature. This is called psychological stress-induced hyperthermia (Adriaan Bouwknecht et al., 2007; Vinkers et al., 2008) and this autonomic stress response is observed in many mammalian species, including humans. Social defeat stress drives the sympathetic thermogenesis in brown adipose tissue that contributes to psychological stress-induced hyperthermia through a glutamatergic neural pathway (Kataoka et al., 2014). Stress-induced hyperthermia is suggested to play an evolutionary adaptive role during the “fight vs. flight response” to threatening situations (Goldstein, 2010). Cohort removal might serve as social and psychological stress, resulting in hyperthermia. Another possibility is that increased locomotor activity in the home cage caused hyperthermia. When mice were removed from their cage, the experimenter searched for the subject animal, which may increase the physical activity and arousal level of all the mice in the cage. Those responses may contribute to the effect of cohort removal. In old mice, cohort removal did not induce hyperthermia compared to young mice (Supplementary Figures 1A,B). The basal serum corticosterone concentration is higher in old mice compared to young mice (Tronche et al., 2010), which may narrow the dynamic range of corticosterone level. Considering those facts, it is reasonable that the response in the old mice was relatively small. This may explain the lack of sensitivity of the old mice to cohort stress-induced hyperthermia.

Cohort removal affected pain sensitivity. The effect did not appear in a stepwise manner. The latency to show a paw response was not significantly different among mice removed second, third, or fourth. Mice removed first exhibited a lower pain sensitivity, however, than those removed later. Considering that pain sensitivity is modulated by stress (Mason, 1999; Imbe et al., 2004), it is possible that cohort removal enhanced pain sensitivity. Although stress affects pain sensitivity, the direction of the influence of stress on pain sensitivity is ambiguous. Chronic restraint stress increases pain sensitivity in rodents (Imbe et al., 2004), while acute restraint stress attenuates pain sensitivity (Imbe et al., 2004). In rodents, social defeat stress also enhances pain sensitivity (Marcinkiewcz et al., 2009). In humans, acute psychological stress decreases pain sensitivity (al’Absi and Petersen, 2003; Reinhardt et al., 2013) and chronic psychological stress enhances pain sensitivity (Caceres and Burns, 1997; Cathcart et al., 2008). Stress-induced enhancement of pain sensitivity, or hyperalgesia, is proposed to represent an increase in vigilance to prevent potential harm by an enemy (Martenson et al., 2009). Cohort removal is not chronic stress, yet it might increase vigilance. This discrepancy may be brought by the difference of the severity of stress. Compared to restraint stress that was used as the acute stress in the previous study (Imbe et al., 2004), cohort removal is apparently mild. Exogenous corticosterone reduces pain sensitivity (Bogdanov and Yarushkina, 2000), but only at a high dose. The increase of plasma corticosterone concentration after the administration was more than 1500 ng/ml (Bogdanov and Yarushkina, 2000). The increase in the plasma corticosterone concentration induced by cohort removal was much smaller than that in the previous reports. These findings suggest that stress-induced hyperalgesia was not due to the upregulation of corticosterone. The mechanisms of enhanced pain sensitivity induced by cohort removal should be further investigated.

In the elevated plus maze test, cohort removal increased the time spent on open arms, entries into open arms, and locomotor activity in group-housed mice. These results suggest that cohort removal affects anxiety-like behaviors. A previous study reported that cohort removal failed to influence anxiety-like behavior in the elevated plus maze test (Rodgers et al., 1994). Rodgers et al. (1994) compared mice among those removed from home cage earlier (positions 1–3), middle (positions 4–7), and later (positions 8–10) which resulted in a lack of a significant effect of cohort removal on anxiety-like behavior. In the present study, we combined our datasets to analyze a large number of mice, and we compared mice among each position of the sequential removal. With our analytical method, we determined that mice removed first displayed higher anxiety-like behavior than mice removed thereafter. In Rodgers et al.’s (1994) study, the mice removed first were included in a group that also contained mice removed second and third, which may dilute the effect of cohort removal on anxiety-like behaviors in an analysis containing the standard (and much lower) number of mice.

Other factors that may cause inconsistencies are differences in the strain, the apparatus, and the protocols. Brodin et al. (1994) reported that cohort removal increases serum corticosterone concentrations in rats when the animals were decapitated in the same experimental room, but did not increase that if the rats were decapitated in another room (Brodin et al., 1994). Our results indicated that cohort removal increased plasma corticosterone concentrations, even if the animals were removed from their cages and transferred to a different room before being decapitated. This corticosterone increase induced by cohort removal may affect behaviors in the elevated plus maze and other tests. However, exposure to the elevated plus maze also increases the plasma corticosterone concentration (File et al., 1994; Rodgers et al., 1999). Corticosterone administration increases the percentage of open arm entries and time spent on the open arms in rats and mice (Andreatini and Leite, 1994). Kalynchuk et al. (2004) also reported that corticosterone injections increase baseline anxiety-like level in rats. In the present study, mice removed earlier had a lower corticosterone concentration and higher anxiety-like behavior in the elevated plus maze, whereas mice removed later had a higher corticosterone concentration and reduced anxiety-like behavior. Considering that phenotypes assessed in the elevated plus maze and light/dark transition tests are not necessarily consistent (Miyakawa et al., 2003; Nagura et al., 2012; Onouchi et al., 2014), time spent on the open arms in the elevated plus maze may not always reflect anxiety in mice. This index might reflect not only anxiety-like behaviors, but also other behavior, such as exploration, escape attempts, or panic reactions. Corticosterone concentrations also reflect the state of wakefulness (Mitsugi and Kimura, 1985) or could be a physical consequence of behavior (Koolhaas et al., 2011). Considering that the experiments were conducted during the light period when nocturnal animals like mice or rats usually sleep, it is possible that cohort removal heightened the arousal state of the mice. A higher corticosterone concentration (Mitsugi and Kimura, 1985; Koolhaas et al., 2011), higher body temperature (Barrett et al., 1993), and increased pain sensitivity (Callahan et al., 2008) could be explained by wakefulness, or as a physical consequence of behavior or higher arousal state.

The effect of cohort removal was not specific to male C57BL/6J mice. In female mice, although the effects of cohort removal were not necessarily significant, there were similar tendencies (Supplementary Figures 1C, 2A, 3A–D). We also conducted the analyses using another substrain. C57BL/6N mice also demonstrated similar tendencies in all three tests (Supplementary Figures 1D, 2B, 3E–H). We also conducted analyses using data from genetically engineered mice (not including wild-type control mice), which resulted essentially the same as found in C57BL/6J mice (data not shown). These findings suggest that cohort removal generally affects rectal temperature, pain sensitivity, and anxiety-like behaviors.

The present study demonstrated a clear effect of cohort removal on the results of several behavioral tests in mice. Because the number of apparatuses for behavioral analyses is sometimes limited, for some tests the mice are tested sequentially using a single apparatus. Consequently, this allows cohort removal to impact the behavior. To evaluate the loss-of-function or gain-of-function effects of a gene of interest, the behaviors of genetically engineered mice are often compared to those of wild-type control littermates. In such a situation, the effect of the position in sequential removal on behaviors could be potentially serious. Therefore, appropriate counterbalancing for the position of sequential removal is essential in conducting behavioral experiments. The order of sequential removal should be counterbalanced across genotypes to minimize the effect of cohort removal on behaviors that are compared between mutant mice and wild-type control mice. Experiments should be carefully designed by considering the effect of cohort removal on the behavior of group-housed animals. When reporting behavioral analyses of rodents, the design of behavioral experiments should include counterbalancing the serial position of removal.

## Author Contributions

KT and TM designed the study. KT, HS, and SH analyzed the data. KT and TM wrote the manuscript.

## Conflict of Interest Statement

The authors declare that the research was conducted in the absence of any commercial or financial relationships that could be construed as a potential conflict of interest.

## Footnotes

^1^http://www.mouse-phenotype.org/

^2^http://rsb.info.nih.gov/nih-image/

^3^http://www.mouse-phenotype.org/software.html

## Supplementary Material

Supplementary Figure 1 **The effect of cohort removals on rectal temperature in young (A), old (B), female (C), and B6N (D) groups.** Female mice consisted of 78 B6J and 40 B6N background mice.

Supplementary Figure 2 **The effect of cohort removals on the latency of the hot plate test in female (A), and B6N (B) groups.** Female mice consisted of 78 B6J and 40 B6N background mice.

Supplementary Figure 3 **The effect of cohort removals on total distance traveled (A,E), total number of arm entries (B,F), time spent on open arms (C,G), and percentage of entries into open arms (D,H) of the elevated plus maze test in female (A–D), and B6N (E–H) groups.** Female mice consisted of 78 B6J and 40 B6N background mice.
