# Supplementary figures and images for: Cohort Removal Induces Changes in Body Temperature, Pain Sensitivity, and Anxiety-Like Behavior
Source: Front Behav Neurosci. 2016 Jun 3;10:99. doi: 10.3389/fnbeh.2016.00099 (PMC4891333; doi:10.3389/fnbeh.2016.00099)

## A Over 200 days old

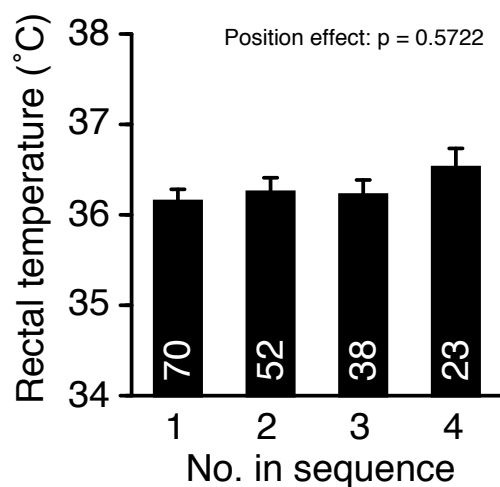

## B Less than 200 days old

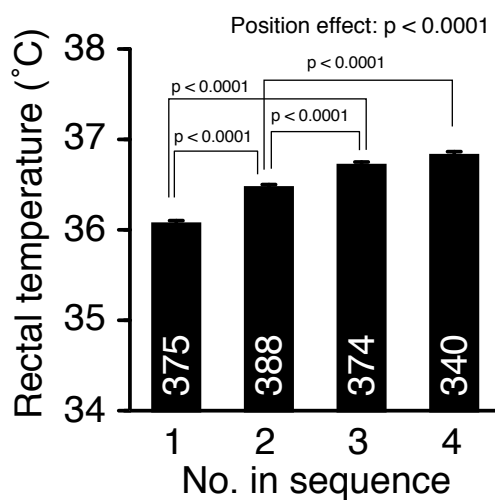

## C Female

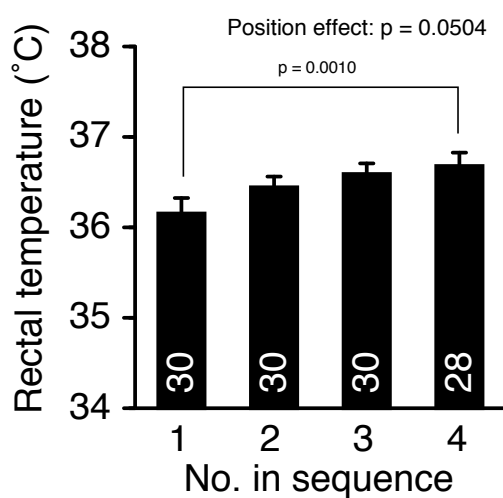

## D B6N

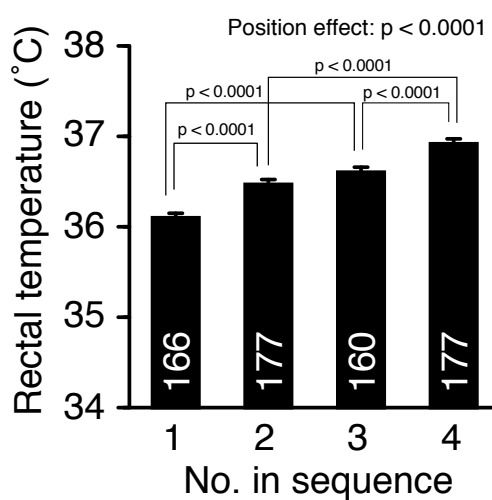

Supp Figure 1

Supplement: Supplementary Figure 1 — The effect of cohort removals on rectal temperature in young (A), old (B), female (C), and B6N (D) groups. Female mice consisted of 78 B6J and 40 B6N background mice. [file Image_1.pdf]

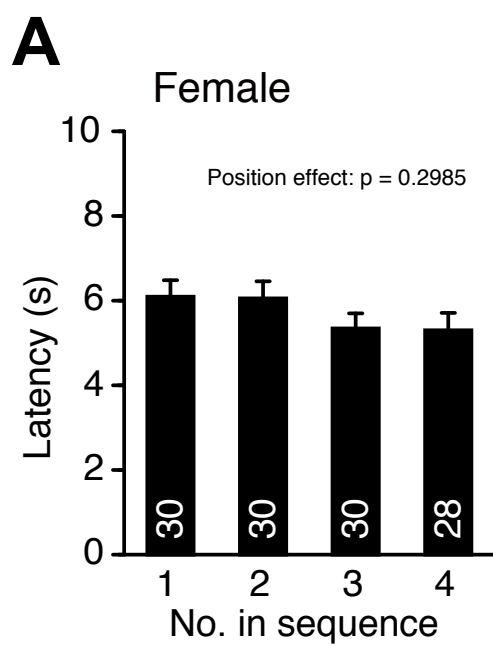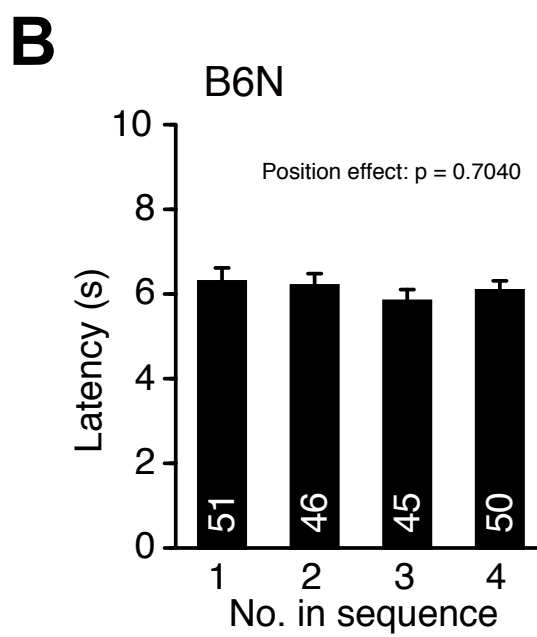

Supplementary figure 2

Supplement: Supplementary Figure 2 — The effect of cohort removals on the latency of the hot plate test in female (A), and B6N (B) groups. Female mice consisted of 78 B6J and 40 B6N background mice. [file Image_2.pdf]

Position effect:  $p = 0.0036$

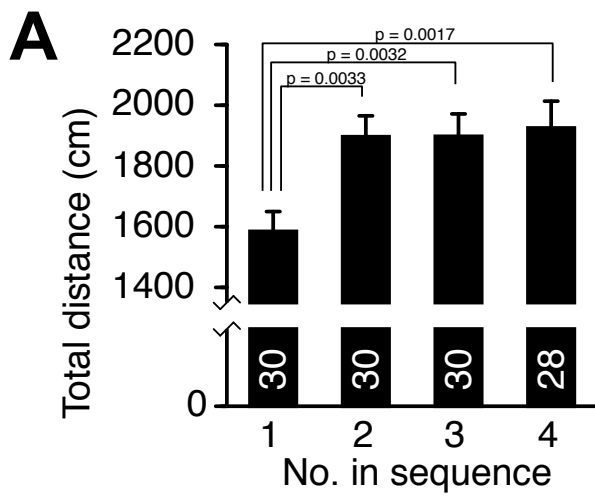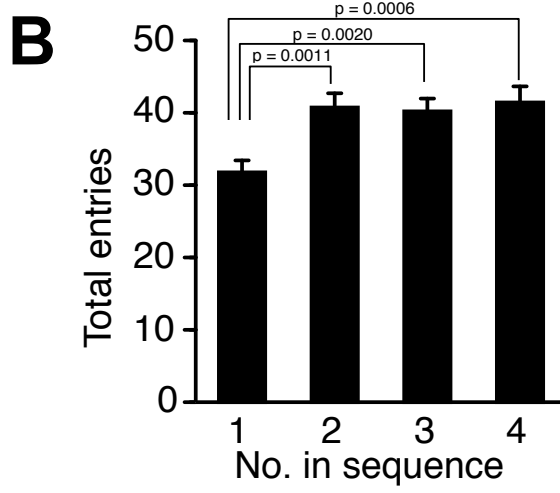

Female

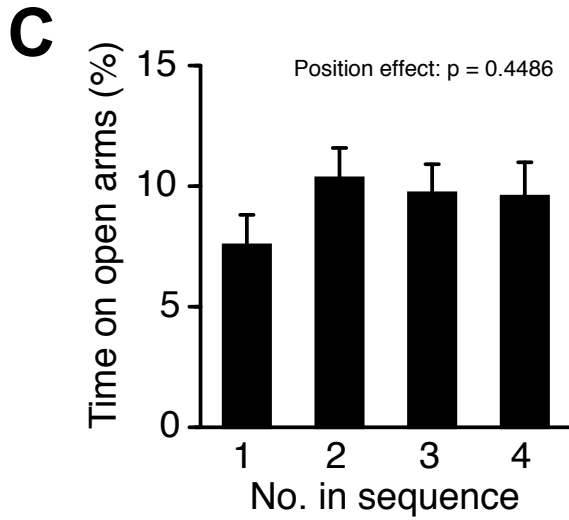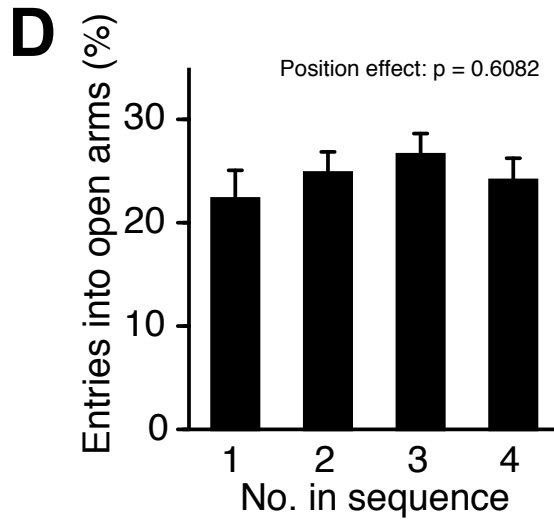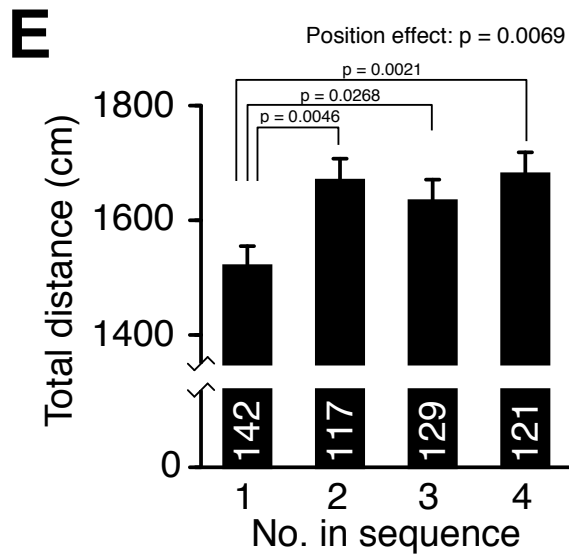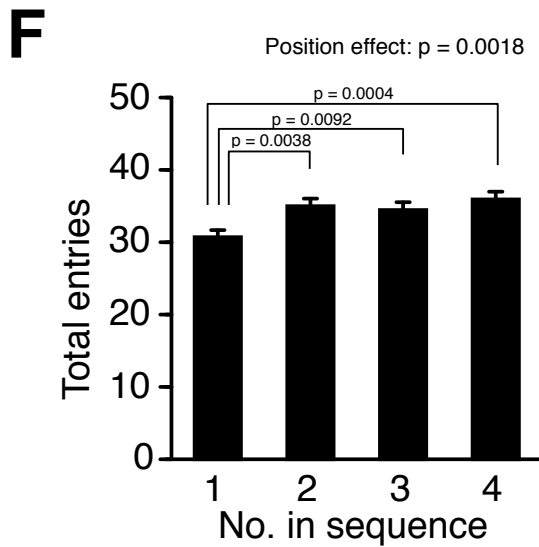

B6N

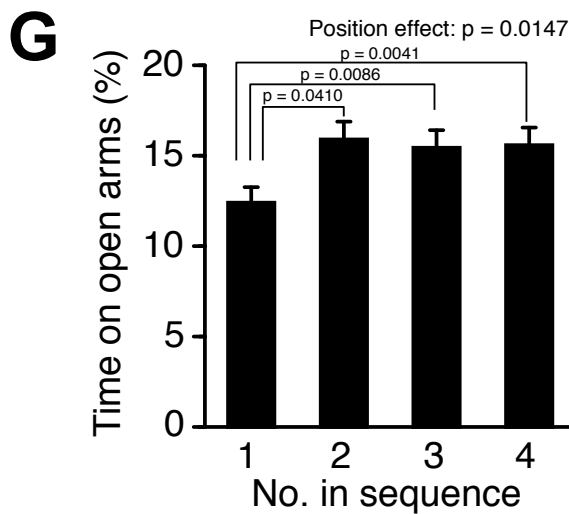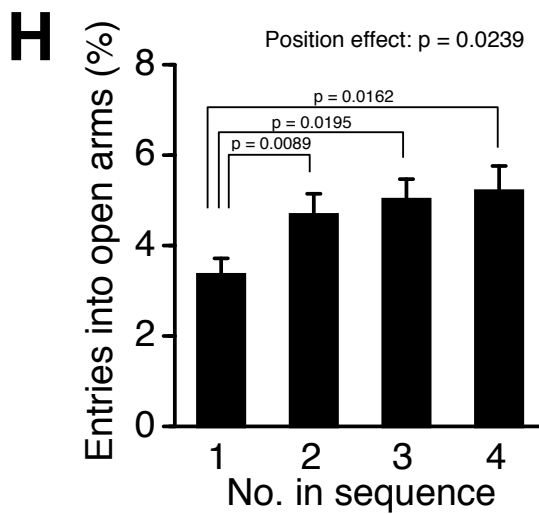

Supplement: Supplementary Figure 3 — The effect of cohort removals on total distance traveled (A,E), total number of arm entries (B,F), time spent on open arms (C,G), and percentage of entries into open arms (D,H) of the elevated plus maze test in female (A–D), and B6N (E–H) groups. Female mice consisted of 78 B6J and 40 B6N background mice. [file Image_3.pdf]
